# Managing the Overlap: Therapeutic Approaches in Patients with Concomitant Psoriasis and Atopic Dermatitis—A Case Series

**DOI:** 10.3390/jcm14030796

**Published:** 2025-01-25

**Authors:** Maria Beatrice de Felici del Giudice, Giorgia Ravaglia, Marco Brusasco, Francesca Satolli

**Affiliations:** 1 Dermatology Unit, Department of Medicine and Surgery, University of Parma, I-43121 Parma, Italy; giorgia.ravaglia@unipr.it (G.R.); fra.satolli@libero.it (F.S.); 2 Dermatology Unit, ASST Santi Paolo e Carlo, I-20142, Milan, Italy; marco.brusasco@asst-santipaolocarlo.it

**Keywords:** atopic dermatitis, psoriasis, PSOREMA, treatment options

## Abstract

**Introduction**: Psoriasis (PSO) and atopic dermatitis (AD) have traditionally been considered distinct diseases, respectively, mediated by T-helper 1 (Th1) and the T-helper 2 (Th2) immune pathway. In recent years, there has been a growing body of evidence highlighting an overlap between the two conditions, such as Asian AD, pediatric PSO, or “psoriasis dermatitis/PSOREMA”. Moreover, psoriasis dermatitis can be induced by therapeutic interventions. For instance, anti-IL-4/IL-13 monoclonal antibodies, commonly used to treat AD, can induce psoriasiform reactions by inhibiting the Th2 pathway, thereby unmasking Th1/Th17-driven PSO. Conversely, anti-TNFα and anti-IL-17 therapies, effective for PSO, may induce eczematous reactions promoting a switch toward Th2-driven inflammation. Janus Kinase Inhibitors (JAK-i) and IL-23 antagonists may represent valid therapeutic options for managing psoriasis dermatitis. JAK-i exert broader immunomodulatory effects, inhibiting both Th1 and Th2 pathways; however, they require careful monitoring due to potential adverse events. In contrast, IL-23 antagonists specifically suppress the IL-23/IL-17 axis inhibiting the p19 subunit of IL-23 and could represent a safer option for patients with psoriasis dermatitis. **Materials and Methods/Results**: We present a series of five cases of psoriasis dermatitis, including both patients who had the condition from the onset and those who developed it during treatment, with tailored therapeutic strategies based on individual patient profiles, comorbidities, and the specific characteristics of their overlapping disease presentation. **Conclusion**: JAK-i and IL-23 antagonists are both valid therapeutic options for managing psoriasis dermatitis, but with different immunomodulatory effects and safety profiles. Future research should focus on a better understanding of the immune pathway and identifying specific biomarkers of psoriasis dermatitis, to optimize therapeutic strategies.

## 1. Introduction

Psoriasis (PSO) and atopic dermatitis (AD) have traditionally been regarded as distinct diseases, each primarily driven by different immune pathways. Specifically, psoriasis is mediated by the T-helper 1 (Th1) and T-helper 17 (Th17) axes, whereas AD involves the T-helper 2 (Th2) pathway. However, recent research has revealed increasing evidence of a significant overlap between these two conditions. This includes clinical presentations such as Asian AD, pediatric PSO, or “psoriasis dermatitis/PSOREMA” [1,2,3]. These overlap syndromes suggest that PSO and AD may not be as immunologically distinct as previously thought, introducing new challenges in treatment. According to a recent review, the prevalence of AD in patients with PSO ranges from 0.17% to 20%, with an overall estimated prevalence of 2%. Similarly, the prevalence of psoriasis in patients with AD ranges from 0.3% to 12.6%, with an overall estimated prevalence of 2% [4]. Managing such cases presents significant challenges, as no therapy has yet been identified that is consistently effective across a broad spectrum of affected patients. The complexity of treatment often depends on the specific characteristics of the patient and their comorbidities. The aim of our case series is to highlight the therapeutic challenges in patients with overlap syndrome and share the best treatment options based on our experience.

## 2. Materials and Methods

We present a case series of five patients with psoriasis dermatitis, each with a tailored therapeutic approach based on individual patient profiles, comorbidities, and the unique characteristics of their overlapping disease manifestations. We collected data from patients referred to our clinic between January 2020 and December 2024 who developed an overlap of PSO and AD and required systemic therapy due to disease severity (Body Surface area, BSA, >10%). In the absence of specific guidelines for managing overlap cases, our therapeutic choices were guided by systemic drugs currently approved for either AD or PSO, selected based on our clinical experience and the individual characteristics of each patient. Moreover, we evaluated the efficacy of therapy through different clinical scores: BSA, Dermatology Life Quality Index (DLQI), and Physician Global Assessment (PGA) (Table 1 and Table 2). For each of the five patients, we obtained informed consent for the collection of personal data and images during routine clinical practice.

## 3. Case Series

**Figure 1:**
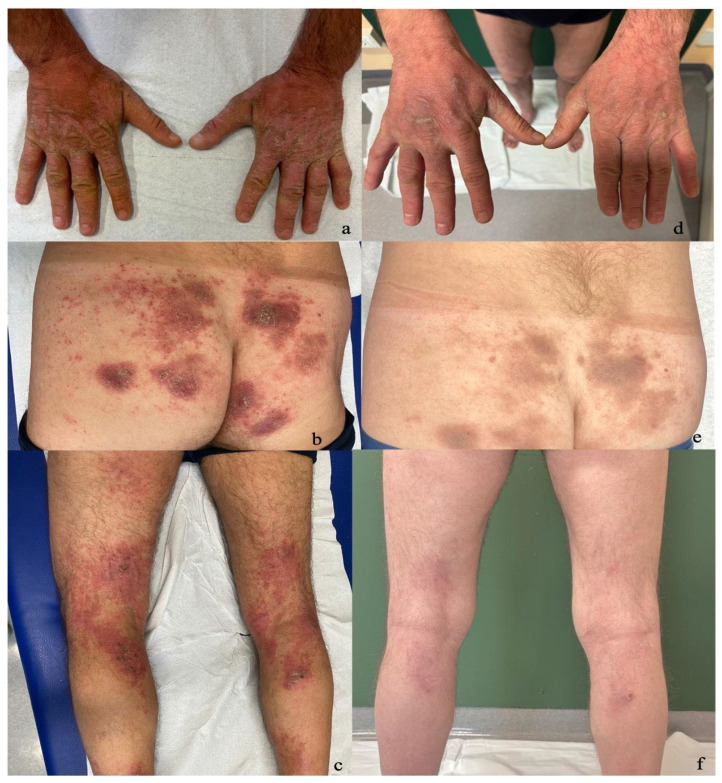
(**a**–**c**) Erythematous-violaceous hyperkeratotic plaques with poorly defined margins localized to the backs of the hands, buttocks, and lower limbs before initiating therapy with risankizumab. (**d**–**f**) Residual hyperpigmentation observed after six months of therapy.

Case 1: A 51-year-old male, without any known comorbidities, was diagnosed with adult-onset AD and was treated with dupilumab from December 2020 until October 2023. However, treatment was discontinued due to the emergence of diffuse erythematous-squamous plaques predominantly localized to the buttocks and lower limbs, including the involvement of the hands (Figure 1a–c). Suspecting an overlap of PSO and AD, a skin biopsy was performed in November 2023, revealing regular psoriasiform hyperplasia of the epidermis, widespread confluent parakeratosis, and hypogranulosis. There was also mild spongiosis with lymphocytic exocytosis. The patient had contraindications for the use of JAK-i due to being a smoker (30 cigarettes a day), so it was decided to initiate treatment with risankizumab 150 mg, administered as a subcutaneous injection every 12 weeks after the induction phase, starting in January 2024. Three months later, the patient showed significant improvement in clinical presentation and subjective symptoms, with a reduction in the PGA from 4 to 2. Additionally, the patient’s BSA decreased from 30% to 6%, and the DLQI improved from 10 to 2. After six months, the patient achieved a PGA of 1, a BSA of 3%, and a DLQI of 2, with residual involvement localized only to the backs of the hands (Figure 1d–f).

**Figure 2:**
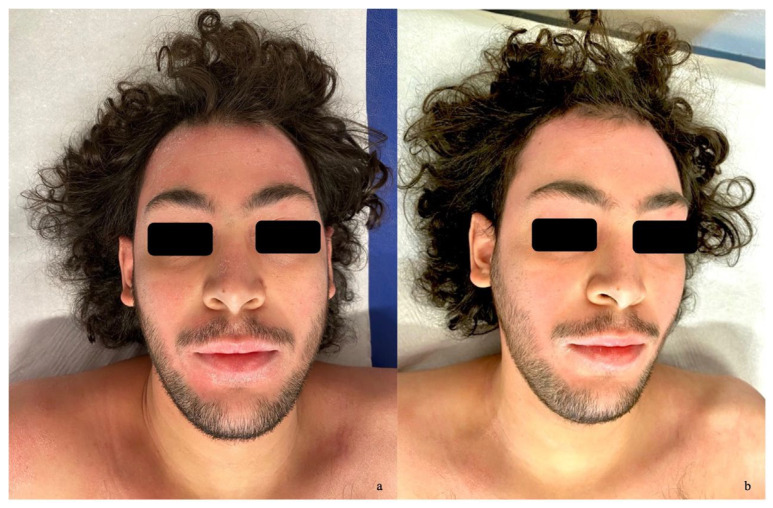
(**a**) Diffuse facial erythema with fine scaling observed before the initiation of therapy. (**b**) Clinical improvement with faint residual erythema after three months of risankizumab.

Case 2: A 23-year-old male presented with a long-lasting history of diffuse atopic dermatitis, dating back to childhood, and a significant family history of atopy (the brother) and psoriasis (the grandmother). In his medical history, the patient was under follow-up for factor VII deficiency. He had been receiving dupilumab since November 2022, but the response was suboptimal. In September 2023, he developed a psoriasiform dermatitis condition involving the face (Figure 2a), upper limbs, and lower limbs. The diagnosis was confirmed by biopsy, revealing psoriasiform hyperplasia of the epidermis with mild spongiosis, foci of parakeratosis, and hypogranulosis. Dupilumab was then discontinued and, considering the factor VII deficiency and a family history of thrombotic thrombocytopenic purpura (the father), it was decided to initiate treatment with risankizumab 150 mg in December 2023, administered as a subcutaneous injection every 12 weeks after the induction phase. After three months of the treatment, the patient showed significant improvement, with a reduction in PGA from 3 to 1, BSA from 15% to 3%, and DLQI from 10 to 2, achieving complete skin clearance at six months (Figure 2b).

**Figure 3:**
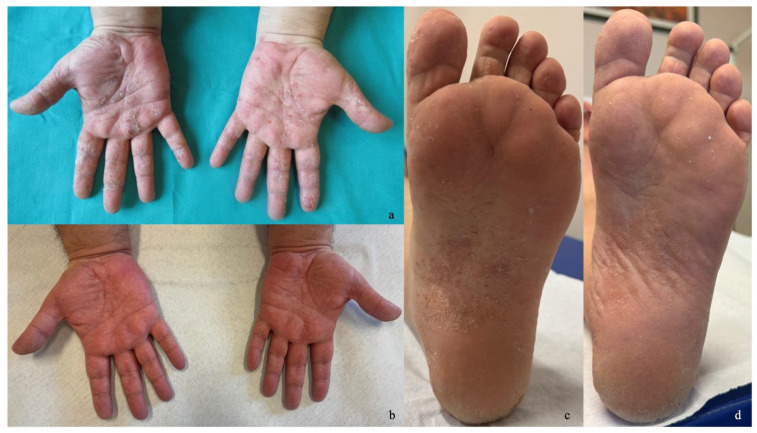
(**a**,**c**) Psoriasiform dermatitis of palms and soles before initiating therapy. (**b**,**d**) Complete resolution of cutaneous manifestations after six weeks of therapy.

Case 3: A 50-year-old male with a personal history of atopy presented to our clinic with eczematous dermatitis on his back in March 2024. In his medical history, the patient had allergic bronchial asthma. The patient was treated with cyclosporine starting in July 2024. By October 2024, while the eczema on his back had improved, he developed a psoriasiform condition affecting the palms and the soles (Figure 3a,c). Given a DLQI >10 and contraindications for JAK-i therapy due to being a smoker (25 cigarettes a day), it was decided to discontinue cyclosporine and initiate treatment with risankizumab 150 mg, administered as a subcutaneous injection every 12 weeks after the induction phase. Six weeks later, the patient showed complete resolution of the eczematous areas on the trunk and an initial improvement in the palmoplantar regions (Figure 3b,d).

**Figure 4:**
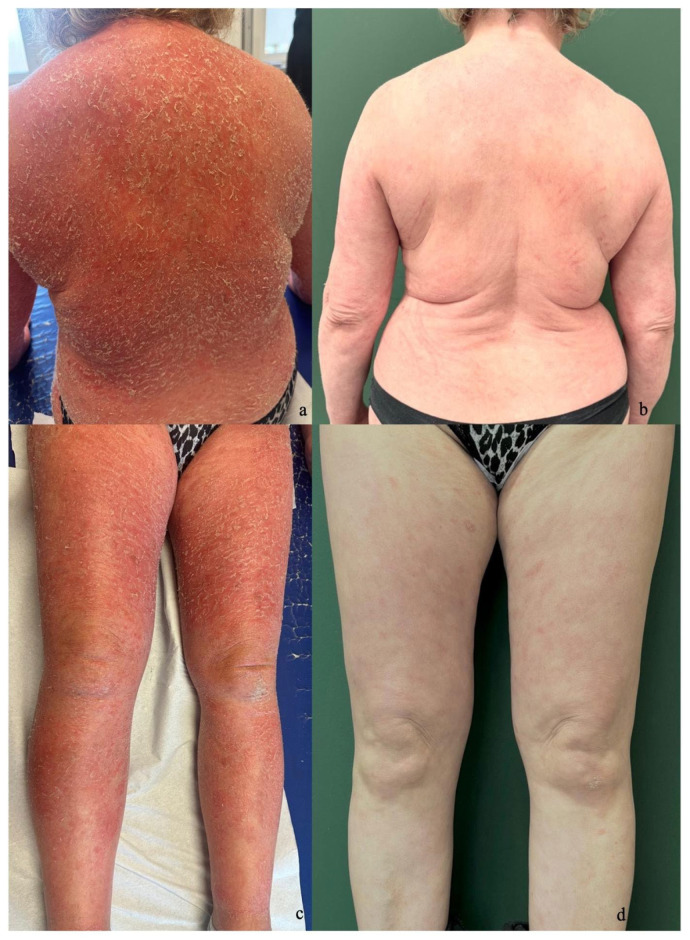
(**a**,**c**) Eczematous erythrodermic dermatitis with lamellar desquamation after two months of bimekizumab treatment. (**b**,**d**) Resolution of dermatitis after three months of abrocitinib therapy.

Case 4: A 62-year-old female was diagnosed with psoriasis in March 2023, presenting with primary lesions on her trunk and face. In her medical history, the patient had arterial hypertension, dyslipidemia, and hypothyroidism. She was subsequently treated with acitretin. Due to the worsening of her condition, she started therapy with tildrakizumab 200 mg in December 2023 and underwent a skin biopsy in January 2024, revealing irregular psoriasiform hyperplasia, hyperkeratosis, parakeratosis, and mild spongiosis. In June 2024, tildrakizumab was discontinued due to the persistence of clinical symptoms, and treatment with bimekizumab 160 mg was initiated. After less than two months, the patient presented to our clinic with erythrodermic eczematous dermatitis (Figure 4a,c). Suspecting that this may be due to the unmasking of atopic dermatitis from anti-IL-17 inhibitors, she received initial steroid treatment and then began therapy with abrocitinib 200 mg once daily. The patient showed significant improvement as early as the first month of treatment, with a BSA reduction from 70% to 8%. After three months of therapy, the BSA had further decreased to 3%, the PGA had improved from 4 before starting abrocitinib to 1, and the DLQI had decreased from 25 to 2 (Figure 4b,d).

Case 5: A 20-year-old female, otherwise healthy, presented with a long-standing history of AD, initially diagnosed in childhood. She was initially treated with dupilumab in November 2023 but subsequently developed significant psoriatic involvement of the scalp, prompting a switch to upadacitinib 15 mg once daily in January 2024. However, due to limited improvement of atopic dermatitis, localized on the face, trunk, and limbs, she discontinued upadacitinib and, in April 2024, started therapy with abrocitinib 200 mg once daily. Remarkably, after three months of treatment, she experienced an improvement in both her scalp psoriasis and body-wide atopic dermatitis, with her BSA decreasing from an initial 20% to 5%, DLQI improving from 15 to 4, and PGA reducing from 3 to 1. By six months, her BSA had further decreased to 3% and her DLQI to 2.

## 4. Discussion

Psoriasis (PSO) and atopic dermatitis (AD) are two conditions with a complex pathogenic basis involving multiple genes. They have traditionally been regarded as distinct diseases, primarily driven by different immune pathways. Specifically, PSO is characterized by the activation of Th1/Th17 cells, whereas AD is predominantly a Th2-mediated disease. Based on the immunological context, when activated, naïve CD4+ T cells differentiate into Th1 cells, which produce chemokines and cytokines including interferon-gamma (IFN-γ), interleukin-17 (IL-17), and tumor necrosis factor-alpha (TNF-α), or into Th2 cells, which produce cytokines such as IL-4, IL-5, and IL-13 [1,2]. These modulatory secretions are highly context-dependent and can ablate, induce, or exacerbate a disease phenotype, depending on a complex interplay of interdependent circumstances.

However, in recent years, growing evidence has highlighted the overlap between these two conditions [2,3,5]. For example, intrinsic AD, which is characterized by normal IgE levels, often shows increased Th1 expression and more pronounced Th17/Th22 activation as seen in Asian AD endotypes and children AD, while Caucasian-American AD is primarily Th2-driven [6,7]. Moreover, a recent study reported that a distinct subtype of psoriasis in the Chinese population exhibits Th2 immune signatures [8]. Thus, AD and PSO may be better understood as part of a disease spectrum rather than as entirely distinct conditions [5]. According to a recent study, the prevalence of overlap cases is approximately 1.3% of all patients diagnosed with either PSO or AD. However, the actual prevalence may be higher, as many mixed clinical cases may be misdiagnosed as only one of the diseases [9].

Recognizing overlap cases can be challenging, but it is essential for informed therapeutic decision-making. This is because certain treatments for AD may unmask or exacerbate PSO, and conversely, some PSO treatments may worsen AD. Blocking the Th2 pathway may result in a shift in T-cell polarization toward Th17 subsets. Similarly, inhibiting the Th1/Th17 pathways may promote the emergence of Th2-mediated conditions. For instance, the use of dupilumab in AD, which targets the IL-4/IL-13 axis, can trigger PSO flares in patients with a prior history of PSO, and in some cases, even induce “de novo” PSO in AD patients with no previous history of PSO [10].

In our case series, three patients with AD undergoing treatment with dupilumab developed “de novo” PSO manifestations, with only one patient having a family history of PSO. Biopsies were performed on two of these patients to confirm the diagnosis, revealing psoriasiform hyperplasia with spongiosis, a histological pattern typical of overlap cases [1].

Interestingly, the first patient of our report developed “de novo” psoriasiform manifestations 34 months after starting dupilumab therapy, whereas the average latency period reported in the literature is 5.6 months, with a range of 1 to 30 months [6].

In our report, four patients initially presented with AD features and later developed psoriatic manifestations, in accordance with some authors who suggest that, in consecutive cases, AD appears to precede the onset of PSO [1]. Notably, two of these patients had significant hand involvement, a common feature in overlap cases [9].

Conversely, one patient with poorly controlled PSO on an IL-23 inhibitor developed erythrodermic AD less than two months after starting anti-IL-17 therapy. This patient likely had an overlap condition from the start, as her biopsy performed before biologic therapy showed psoriasiform hyperplasia with mild spongiosis. However, the initial clinical presentation appeared to be compatible with plaque psoriasis, which led to treatment with standard biologics for PSO.

From a therapeutic perspective, treatment options for patients with PSO-AD overlap remain limited, as most available drugs are designed to target either eczema or PSO, but not both conditions simultaneously. Traditional immunosuppressive therapies (such as cyclosporine and methotrexate) and phototherapy are unsuitable for long-term use due to safety concerns, and many selective cytokine inhibitors often show limited effectiveness. In fact, some treatments, such as dupilumab, may even contribute to the onset of psoriasiform manifestations in certain patients. This treatment gap underscores the need for therapies that can effectively address both conditions in cases of overlap.

Recent studies suggest that upadacitinib may be an effective treatment option for patients with overlapping PSO and AD [11,12]. JAK-i exert broader immunomodulatory effects, inhibiting both Th1 and Th2 pathways, and are currently indicated for many immune-mediated conditions, including dermatologic, hematologic, rheumatologic, and gastroenterological diseases [13]. In patients with overlapping PSO and AD, the inhibition of the JAK pathway may help manage the condition, given its role in both the IL-23/IL-17 inflammatory axis and Th2 pathway upregulation. In our case series, JAK-i were used in two patients: one with an erythrodermic presentation, who showed rapid improvement with abrocitinib within the first month, and another who had a good response for scalp PSO with upadacitinib 15 mg (the only dosage approved in Italy at that time) but only a partial response for body AD, which later fully resolved with abrocitinib. In accordance with the literature and as seen in our case series, JAK-i, currently approved in Italy for AD, have shown efficacy in overlap cases. However, they require careful monitoring due to potential adverse effects, especially in patients with cardiovascular comorbidities or conditions associated with increased blood clotting risk [14]. In our report, two patients were heavy smokers (more than 20 cigarettes a day), and one had hematological comorbidities, making the use of JAK-i unsafe. Based on recent studies indicating that patients with PSO-AD overlap often exhibit a genomic profile typical of classic PSO, characterized by the overexpression of Th17- and Th1-related cytokines, we opted for treatment with risankizumab, an IL-23 inhibitor [5,15]. The selective blockade of the p19 subunit of IL-23 results in the upstream downregulation of the IL-23/IL-17 pathway, reducing the production of multiple inflammatory cytokines acting on keratinocytes. Safety data suggest that IL-23 inhibitors are less likely to induce atopic-like eczema compared to anti-IL-17 therapies [1]. In our case series, two patients treated with risankizumab demonstrated significant improvements, with a ≥2-point reduction in the PGA score at three months, and their DLQI improved from 10 to 2. Specifically, one patient showed a reduction in baseline BSA involvement from 15% to 3% at three months and achieved complete resolution at six months (BSA 0%, PGA 0, and DLQI 0), with this outcome maintained throughout follow-up.

The other patient experienced a reduction in BSA from 30% to 6% at three months, further improving to 3% after six months. The third patient began risankizumab more recently, and after one and a half months, we observed an initial improvement, with the hand PGA decreasing from 3 to 2 and we continue to carefully monitor further progress. From our clinical experience, IL-23 inhibitors could represent a valid alternative to JAK-i in certain patients who present risk factors that limit therapeutic options.

## 5. Conclusions

The overlap between PSO and AD represents a paradigm shift in dermatology, challenging clinicians to move beyond traditional disease definitions and adopt a more nuanced approach to immune-mediated skin disorders. As our case series demonstrates, identifying cases of overlap is essential for making informed therapeutic decisions. The efficacy of IL-23 inhibitors and JAK inhibitors in our series highlights their potential in managing these complex syndromes, with IL-23 inhibitors emerging as a valuable alternative for patients with significant cardiovascular comorbidities, where JAK inhibitors may pose risks. Our findings emphasize the need for a flexible, individualized therapeutic approach, tailored to the unique immune profile and comorbidities of each patient. However, our experience is limited due to the small sample of patients and the relatively short follow-up, which does not allow us to assess the long-term effectiveness of the therapy. Studies involving larger patient cohorts are needed to better define therapeutic indications for the management of complex cases. As knowledge of the immune pathways underlying the PSO-AD overlap continues to advance, future research should focus on identifying specific biomarkers that can guide personalized treatment strategies and optimize clinical outcomes.

## Figures and Tables

**Table 1:** Case series of psoriasis dermatitis.

Patient	Age and Sex	Initial Clinical Suspicion	IgE (U/L)	Smoker	BMI	Comorbidities	Biopsy	Prior Treatments	Time Before PSO-AD Onset (Months)	Body Site Involved
CASE 1	51 M	AD	48	yes	22	None	yes	Cyclosporine Dupilumab	34	Hands, buttocks, and upper and lower limbs
CASE 2	23 M	AD	117	yes	23	Factor VII deficiency	yes	Dupilumab	10	Face and upper and lower limbs
CASE 3	50 M	PSO	>1000	yes	-	Allergic bronchial asthma	no	Cyclosporine	7	Back, palms, and soles
CASE 4	62 F	PSO	-	no	26	Arterial hypertension Dyslipidemia Hypothyroidism	yes	Acitretin Tildrakizumab Bimekizumab	2	Erythrodermic
CASE 5	22 F	AD	-	no	-	None	no	Dupilumab Upadacitinib	2	Scalp, face, trunk, and upper and lower limbs

AD: atopic dermatitis; BMI: Body Mass Index; PSO: psoriasis; PSO-AD psoriasiform dermatitis.

**Table 2:** Treatment and clinical outcome.

Patient	Current Therapy	Duration of Follow-Up (Months)	Clinical Score	Baseline	T1 (After 1 Month)	T2 (After 3 Months)	T3 (After 6 Months)
CASE 1	Risankizumab 150 mg	11	PGA	4	-	2	1
			BSA	30	-	6	3
			DLQI	10	-	2	2
CASE 2	Risankizumab 150 mg	12	PGA	3	2	1	0
			BSA	15	6	3	0
			DLQI	10	3	2	0
CASE 3	Risankizumab 150 mg	2	PGA	3	2	-	-
			BSA	8	4	-	-
			DLQI	11	8	-	-
CASE 4	Abrocitinib 200 mg	4	PGA	4	2	1	-
			BSA	70	8	3	-
			DLQI	25	5	2	-
CASE 5	Abrocitinib 200 mg	8	PGA	3	3	1	1
			BSA	20	10	5	3
			DLQI	15	15	4	2

BSA: Body Surface Area; DLQI: Dermatology Life Quality Index; PGA: Physician Global Assessment.

## Data Availability

The original contributions presented in this study are included in the article. Further inquiries can be directed to the corresponding author.

## Abbreviations

The following abbreviations are used in this manuscript:

PSO	Psoriasis
AD	Atopic dermatitis
PSO-AD	Psoriasiform dermatitis
Th-1	T-helper 1
Th-2	T-helper 2
JAK-i	Janus Kinase Inhibitors
PGA	Physician Global Assessment
BSA	Body Surface Area
DLQI	Dermatology Life Quality Index
